# Reflections on the immunology of tuberculosis: will we ever unravel the skein?

**DOI:** 10.1186/1471-2334-14-S1-S1

**Published:** 2014-01-08

**Authors:** Maurizio de Martino, Luisa Galli, Elena Chiappini

**Affiliations:** 1 Department of Health Sciences, Meyer Children University Hospital, University of Florence, Florence, Italy

## Abstract

Many and large dumps exist in our knowledge about M*ycobacterium tuberculosis* infection and disease in infants and children. We still do not understand why some individuals do acquire and others do not acquire the infection in the presence of the same risk factors. We do not understand why some individuals convert from latent to active tuberculosis and why other individuals convert from active to inactive tuberculosis even without treatment.

As a matter of fact the immune system mounts a bouncing, robust and polyedral defence against *Mycobacterium tuberculosis*, but the bacillus is so much artful and dextrous that it has ahead from this immunological fierce accoutrements. *Mycobacterium tuberculosis* survival, multiplication, and transmission are largely favoured by the immune mechanisms. The granuloma itself is more *bacillus*- than host-protective.

These abilities make *Mycobacterium tuberculosis* one of more successful human pathogens, but dumps in our knowledge and the counterproductive immunity hinder development of new diagnostics, therapies and vaccines. This occurs in front of an infection which engages one third of the world population and a disease which kills in a year about 1.5 million individuals worldwide.

Understanding mechanisms and meaning of immune response in tuberculosis marks out the foundations of strategies with a view to prepare effective vaccines and reliable diagnostic tools as well as to build up therapeutic weapons. To gain these objectives is vital and urgent considering that tuberculosis is a common cause of morbidity and is a leading cause of death.

## 

Tuberculosis is a paradox disconcerting for immunologists since a good immune response is developed in most individuals, but this response does not just eliminate the infection. On the contrary it aids the survival of *Mycobacterium tuberculosis*, assists its replication and transmission and induces the bacterium to adopt a silent underhand state from which it can reactivate as it pleases [1-4].

In addition, we would like to understand why some individuals do acquire whilst others do not acquire the infection (exposure conditions being equal), why some rare individuals get rid of infection, and how some individuals (without any treatment) are able to convert an active into an inactive infection [5].

## In spite of innate immunity, *mycobacteria* organize their bridgehead

Paraphrasing a famous aphorism of general Erwin Rommel, with regard to the amphibious warfare, but well appropriate in the contest of tuberculosis, *all is decided the first day*, *which gets the longest day*.

Sometimes, soon after inhalation of *Mycobacterium tuberculosis*, bacilli are phagocitized by alveolar macrophages which can kill them and settle the case. This probably depends the coincidence of lucky factors including an intrinsic capacity of macrophages to generate reactive oxygen intermediates (ROI) and consequent microbicidal activity, a favorable inflammatory environment at the infection site, and not outstanding pathogenetic abilities of the inhaled strain [4].

If bacilli survive during the *longest day* (this is the commonest occurrence) they have won the fight. They proliferate at a logarithmic rate within dendritic cells and alveolar macrophages inducing the production of proinflammatory cytokines including interleukin (IL)-1-α, IL-1β, tumor necrosis factor *Ianus Bifrons*, IL-6, IL-12, and interferon (IFN)-γ [2-4].

The role of these cytokines calls to mind the myth of *Ianus bifrons:* IFN-γ regulates T-cell response to mycobacterial infection, promotes antigen presentation, activates phagocytosis, production of ROI, TNF-α mediates early inflammatory responses against infectious agents also stimulating IL-1 and IL-6 production. The susceptibility to mycobacterial infection of TNF-α deficient the mice (which is unable to undertake the formation of granuloma: but, it desirable or not? See afterwards), the increased susceptibility to *bacillus* of Calmette and Guérin (BCG; formerly *vaccin Bilié de Calmette et Guérin*) infection in mice who received anti-TNF-α antibodies and the mycobacterial susceptibility of human treated with TNF-α blockers should suggests its protective role. Also IL-6 is fundamental in developing the early inflammatory response. Its absence in mice may inhibit the maintenance of the protective (may be) IL-17 and causes a delayed IFN-γ production and consequently determines an increased mycobacterial burden. It is well known that IL-12 promotes the TH-1 response and that its deficiency is associated in humans with an increased susceptibility to mycobacterial infection [3,4,6,7]

This first immune mechanism is largely mediated by molecules of the innate immunity. A large number of them are involved, including multiple pattern recognition receptors (that is toll-like receptors), C-type lectin receptor family, cytosolic pattern-recognition receptors, pyrin domain containing 3, and nucleotide-binding oligomerization domain protein 2. These molecules recognize as many again a number of mycobacterial products such as lipoproteins, lipomannans, and DNA [8-12].

The main consequence of recognition of mycobacterial molecules by innate immunity molecules is not only the expression of pro-inflammatory cytokines, but also the expression of cellular adhesion molecules and chemokines which mobilize and activate macrophages, dendritic cells, and neutrophils [11].

In many other infectious diseases this activity should be considered commendable and useful [1]. Instead, in tuberculosis, the inflow of these phagocytic cells supplies a lot of cellular niches that allow the *mycobacteria* to grow without hindrance [1,13,14]. The mycobacterial population grow and spread to freshly recruited adjacent uninfected cells. This is preceded (admirable strategy) by a prolonged survival of the infected cell caused by the *Mycobacterium tuberculosis* itself. In fact, Mycobacterium tuberculosis inhibits the cell apoptosis also allowing a larger number of bacteria are accumulated before bacteria go out of the dead cell [15]. By means of this mechanisms, the induction of the adaptive immunity is delayed [15]. Also cell death is regulated by the *Mycobacterium of ttuberculosis*. In fact, the virulence factor ESX1 type VII secretion system allow the bacteria to induce cell death, allowing them a well-timed exit [16]. It is well known that *Mycobacterium bovis* (used in the BCG vaccine) is devoid of the ESX1 type VII secretion system [17].

In few words: during this first stage of the immune response managed by the innate immunity, the *Mycobacterium tuberculosis* overcomes in big styles.

## The armed equilibrium during the adaptive immunity phase

Whereas the adaptive immunity to influenza virus is recruited after 20 hours, innate immunity against tubercular infection is activated after more than a 40 days. Likely, this delay is due to the delayed transport by dendritic cells of live *mycobacteria* from the lungs to the draining lymphonodes [1,18]. This delayed transport is caused by the inhibition of transport (which should be stimulated by ligands for the CC-chemokine receptor 7) mediated by *Mycobacterium tuberculosis *[18]. The activation of the adaptive immunity, with an accumulation of effector CD4^+^ and CD8^+^ T-cells in the lungs, open a period of trench warfare during which the bacterial load remains stable. Thus the adaptive immunity is not able to eliminate the infection but not even to reduce its entity. Since during this period of apparent immunological equilibrium *mycobacteria* accumulate mutations and a subset of bacteria insist to replicate [1].

Some of the mechanisms which cause this defeat are proper to immune cells and include expansion of Foxp3-expressing regulatory T-cells, defective antigen recognition, and reduced macrophage major histocompatibility complex expression and antigen processing [19]. Other mechanisms are proper to the *Mycobacterium tuberculosis* and include the resistance to the activation of macrophage function mediated by IFN-γ and blinding of specific CD4^+^ T-cell receptors caused by the downregulation of some genes which determine the vanishing of sensible microbial antigens. During this period, nitric oxide, carbon monoxide, and hypoxia predominate in the infection microenvironment causing the expression of a regulon (controlled by the signal transduction system DosR-DosS) which allows *Mycobacteria* use lipids as an alternative energy source [20].

During the adaptive immunity phase phase, a balance between T_H_1 (and consequent IFN-γ production) and T_H_17 (and consequent IL-17 production which causes neutrophil recruitment and tissue damage) cells can control bacterial growth and limit immunopathology [21].

During this phase γδ T-cells play a significant defensive role [22]. *Mycobacterium tuberculosis* activate *γδ* T-cells but also infected monocytes are efficient accessory cells for *γδ* T-cells in a non-major histocompatibility complex-restricted also by means of IL-15. Analogously to *αβ*T-cell receptor^+^, *γδ* T-cells optimize their function interacting with adhesion molecules and other molecules including CD40-CD40L, CD28-B7.1/7.2. *γδ* T-cells produce both TNF-*α* and IFN-*γ.* Release of IFN-*γ* is more efficient that that from CD4^+^ T-cells.

During this second phase, *Mycobacteria* do not gain ground, but gain a crucial strategic victory: they preserve and reinforce their bridgehead. Thus far the infected subject has the latent tuberculosis. One third of the world's population have been infected with M*ycobacterium tuberculosis* and have this form of tuberculosis [23].

## The attack: from latent to active tuberculosis

At the right moment (right by the point of view of *Mycobacterium tuberculosis*) the resuscitation-promoting factor may be activated, even years and years after initial infection, resuscitating bacteria from the nutrient-starved state and determining the establishment of an active symptomatic disease. Sometimes (especially in areas with a high prevalence of infection) active disease is determined by re-infection with a second strain. The latent infection turn into an active infection. This change may be due to *mycobacteria* as well as to the host [1].

Differently from viruses (which have fixed programs of expression of their genes), *mycobacteria* are able to change expression of their genes according to necessities and stimuli which are derived from the environment. Thus *mycobacteria* can change the expression of their surface antigens and evade T-cell recognition. In mice *mycobacteria* persist in the lungs also because, as soon as CD4^+^ and CD8+ T-cells enter the lungs, ESAT6 and Ag85B antigen expression is downregulated [24].

The host counterpart of re-activation may be an immunological low on guard [25]. Mechanisms are very poorly understood. Some model exist, including anti-TNF monoclonal antibodies used in a number of autoimmune disease, treatments with steroids, and the CD4^+^ loss in patients with human immunodeficiency virus-type 1 (HIV-1) infection . Interestingly, re-activation in HIV-1 patients occurs largely before the nadir of CD4^+^ cells, because HIV-1 selectively eliminates *Mycobacterium tuberculosis* antigen-specific CD4^+^ cells at a higher rate than other antigen-specific CD4^+^ cells.

It is possible that this is a more general mechanism of T-cell exhaustion in tuberculosis since the mycobacterial load in the lungs is inversely related to the proportion of blood antigen-specific T-cells. In addition, unfavourable polymorphisms of chemokines may alter the cell trafficking in the lungs a reduce the recruitment of cells which should contain the infection. It has been reported that some patients with latent TB have signatures similar to those in patients with active TB. They manifest a specific 86-transcript active TB. This signature is dominated by a neutrophil-driven IFN-inducible gene profile, consisting of both IFN-γ and type I IFN-αβ signalling [26]. These findings underscore the role of IFNs and neutrophils in the development of active tuberculosis

More vague are other possible mechanisms [1]. One is the supposed longer delay in the development of adaptive immunity (observed in the diabetic mice and possibly caused by difficulties in the movement of dendritic cells from the lungs to the lymph nodes) which could explain the increased frequency of re-activation in the diabetic patient. It is an old story that thinnish people (besides their nutritional status) are more prone to tubercular re-activation. This association may depend on leptin which regulates, besides appetite, also growth and activity of T_H_1 cells.

As a proof that this second stage of immune response managed by adaptive immunity is a dynamic process of shooting war, the patients may present fever and *erythema nodosum *[27].

## Only sometimes immunity holds on tightly

Even though the mechanisms are unexplored, it is known that active infection is not a single track. In fact, a substantial but anyway minority portion of patients with active tuberculosis can succeed in getting rid of infection and lead to an inactive tuberculosis. Whereas patients with latent tuberculosis do not manifest chest-X-rays findings, subjects with inactive tuberculosis have chest-X-rays changes [1].

Memory T cells are distinguished by function and migratory capacity. Central memory T-cells have high proliferative activity and produce IL-2, whereas effector memory T-cells produce bothIL-2 and IFN-γ. Circulating effector memory T-cells indicates that an antigen-specific response persists, whereas circulating central memory T-cells indicate that infection has been controlled [28].

Subjects with inactive tuberculosis evidence CD4^+^ central memory T-cell response. By contrast, patients with active tuberculosis evidence CD4^+^ effector memory T-cell responses [29].

## *Mycobacteria* flood

The ultimate objective of all living beings is to disseminate as much as possible their genetic stock. It could seem unbelievable, but the adaptive immunity does favour the transmission of *mycobacteria *[1].

For transmission *mycobacteria* need a large surface exposed to the exterior. This surface allows the *mycobacteria* are expelled by an infected (and coughing) patient with active tuberculosis, reach and infect a previously uninfected individual [30,31]

A large surface is obtained by means the development of cavitary tuberculosis which is the result of lung tissue destruction. There are several lines of evidence, deriving from the HIV-1 infection model, suggesting that T-cells contribute to the development of cavitary tuberculosis: 1) HIV-1 infected subjects less frequently have cavitary tuberculosis; 2) HIV-1 infected patients less frequently transmit *mycobacteria* as compared to HIV-1 uninfected people; 3) in HIV-1 infected patients more the CD4^+^ T-cells and more the frequency of cavitary tuberculosis. Mechanisms are unknown. In very general terms it can be supposed that T-cells promote a high level of inflammation which damage lung tissue [32].

As a matter of fact, strains of *Mycobacterium tuberculosis* diverged genetically many and many years ago (to give an idea: during the upper paleolithic, roughly at the end of the last ice age), but all strains have conserved epitopes which are just that recognized by T-cells [33]. When something is preserved by a species for a long time during evolution it means that it is fundamental for survival of the species. From this we may conclude that for *Mycobacterium tuberculosis* it is an evolutionary advantage to be recognized by T-cells [34,35].

## Granulomata: friend or foe?

Richard Morton descovered in 1679 granuloma (at the time termed tubercles) and described it in his book entitled Phthisiologia more than two centuries before the discovery of the agent causing tuberculosis [36].

Epithelial granulomata are the hallmark of tuberculosis. For a long time it has been believed that these evolutionarily primitive structures control the infection since they wall out bacilli. Unfortunately, it is not so [37-39].

An (apparently) hardened and dynamic arsenal of well organized immunity cells is concentrated in tubercular granulomata. T-cells, B-cells, neutrophils, fibroblasts, macrophages (infected or uninfected), epithelioid cells derived from macrophages, foamy macrophages, and multinucleated giant Langehans cells make up the tubercular granulomata. Granulomata development starts shortly after infection. At the beginning an innate granuloma develops which is composed by neutrophils and alveolar macrophages. Unfortunately, *mycobacteria* within granulomata are able to disarm alveolar macrophages arresting the phagolysosome fusion thus blocking the killing of internalized *mycobacteria*. *Mycobacteria* also drive in infected and harmless (by the point of view of *mycobacteria*) macrophages the production of chemoattractant and pro-inflammatory cytokines. In addition, the relase by *mycobacteria* of the 6 kDa early secretory antigenic target causes the activation of the epithelium which stimulates the recruitment of macrophages causing the expression of matrix metalloproteinase-9 (MMP-9). Also the expression of MMP-1 is driven by *mycobacteria *[40-42]

The result of this frantic fascination towards immune cells is the development of the immune granuloma and the multiplication of fresh *pabulum* for mycobacteria. Mechanisms involved in the promotion and development of granulomata are mostly driven by mycobacteria and this clearly suggests that all is done in the mycobacteria's own interest [42].

TNF is believed to be fundamental for defence against *Mycobacteria* and for granuloma formation. Howeever, its iatrogenic inhibition disrupts granuloma allowing replication of mycobacteria and thus increases the efficacy of chemotherapics [43].

Thus, contrary to the traditional view of a protective role of granulomata, it is now convincing that granulomata favour the persistence and diffusion of the *mycobacteriun* more than its segregation or even its elimination.

## B-cells and antibodies: intelligence agents, ancillary services, or useless?

The role of humoral immunity in tuberculosis is, at the very least, evanescent. On the one hand there are the inconsistent findings in studies of passive immunization, the irrelevance of humoral deficiencies and B-cell immune defects in *Mycobacterium tuberculosis* infection and disease and the ambiguous results in knockout mice besides the general rule that antibodies are nearly ineffective against intracellular agents [44-47].

On the other hand, there are well known exceptions to this rule (infections caused by species of *Leishmania*, *Chlamydia*, *Francisella*), the levels of specific antibodies correlate to the efficacy of BCG vaccine, mice knockout for polymeric immunoglobulin receptors have a higher susceptibility to *mycobacterial* infections (is there a role of mucosal dymeric IgA?), in animal models monoclonal antibodies against *mycobacterial* antigens have a beneficial effect in tuberculosis. In addition, there is evidence that B-cells are a fundamental component of granulomata and follicle-like B-cell aggregates (site of antigen presentation to T-cells?) are a characteristic feature of granulomatous progession in mycobacterial infection.

In a broader view it is well known that B-cells and humoral immunity collaborate with T-cells and cellular immunity in almost all infectious diseases and B-cells act as antigen-presenting cells to T-cells.and/or polarize T-cell activity influencing the production of cytokines. B-cells are deeply involved in the production in the lungs of the anti-inflammatory IL-10.

It is a peculiar finding that sometimes these mechanisms work in the presence of B-cells, but in the absence of antibodies. Sometimes the opposite occurs. Even more singularly, in tuberculosis and particularly in active tuberculosis there is an significantly elevated prevalence of subjects with autoantibodies [48].

## Conclusions

Tuberculosis is a major international public health. Great efforts are being made by science to oppose this plague. But, candidly, translational results are still stalling.

Each human (and his/her immune mechanisms) is very similar but also largely differ from other humans (and their immune mechanisms). Maybe that personalized medicine is the way to unravel the skein. Results of how immune gene polymorphisms and, in particular, polymorphisms concerning how toll-like receptor and autophagy-related genes influence tuberculosis susceptibility and clinical course are consistent with line of thinking [49-51]. Maybe that some immunological dogma should be revised. One of many is the ambiguous role of IFN-γ [52]. Maybe that some branch of research should be implemented. One is that concerning the unaccountably underestimated role of γδ T-cells [22]. Maybe that, if we want to win the fight against *Mycobacterium tuberculosis*, we must renounce to conformism. It is time for a different way of thinking.

## Abbreviations

BCG: bacillus Calmétte Guerin; IFN: interferon; IL: interleukin; MMP: metalloproteinase; ROI: rective oxygen intermefiate; TNF: tumor necrosis factor.

## Competing interests

the authors declare that they have no competing interests.

## Authors' contributions

MdM conceived the idea, collected data from the literature, and drafted the manuscript. EC and LG helped to draft and critically reviewed the manuscript.

## Declarations

Funding for this supplement has come from a grant of the Italian Health Ministry /Young Research Project.

This article has been published as part of *BMC Infectious Diseases* Volume 14 Supplement 1, 2014: Highlights in Pediatric Tuberculosis. The full contents of the supplement are available online at http://www.biomedcentral.com/bmcinfectdis/supplements/14/S1
